# Enzyme-catalyzed synthesis of selenium-doped manganese phosphate for synergistic therapy of drug-resistant colorectal cancer

**DOI:** 10.1186/s12951-023-01819-0

**Published:** 2023-03-01

**Authors:** Manman Pei, Kaiyuan Liu, Xiao Qu, Kairuo Wang, Qian Chen, Yuanyuan Zhang, Xinyue Wang, Zheng Wang, Xinyao Li, Feng Chen, Huanlong Qin, Yang Zhang

**Affiliations:** 1 School of Medicine, Anhui University of Science and Technology, 168 Taifeng Street, Shannan New District, Huainan, 232000 Anhui Province People’s Republic of China; 2 Nanotechnology and Intestinal Microecology Research Center, Shanghai Tenth People’s Hospital, School of Medicine, Tongji University, 301 Yanchang Road, Shanghai, 200072 People’s Republic of China; 3 Precision Medicine Center, Taizhou Central Hospital, 999 Donghai Road, Taizhou, 318000 Zhejiang Province People’s Republic of China

**Keywords:** Colorectal tumor, Multidrug resistance, Chemodynamic therapy, Enzyme-catalyzed synthesis, Synergistic therapy

## Abstract

**Background:**

The development of multidrug resistance (MDR) during postoperative chemotherapy for colorectal cancer substantially reduces therapeutic efficacy. Nanostructured drug delivery systems (NDDSs) with modifiable chemical properties are considered promising candidates as therapies for reversing MDR in colorectal cancer cells. Selenium-doped manganese phosphate (Se-MnP) nanoparticles (NPs) that can reverse drug resistance through sustained release of selenium have the potential to improve the chemotherapy effect of colorectal cancer.

**Results:**

Se-MnP NPs had an organic–inorganic hybrid composition and were assembled from smaller-scale nanoclusters. Se-MnP NPs induced excessive ROS production via Se-mediated activation of the STAT3/JNK pathway and a Fenton-like reaction due to the presence of manganese ions (Mn^2+^). Moreover, in vitro and in vivo studies demonstrated Se-MnP NPs were effective drug carriers of oxaliplatin (OX) and reversed multidrug resistance and induced caspase-mediated apoptosis in colorectal cancer cells. OX@Se-MnP NPs reversed MDR in colorectal cancer by down-regulating the expression of MDR-related ABC (ATP binding cassette) transporters proteins (e.g., ABCB1, ABCC1 and ABCG2). Finally, in vivo studies demonstrated that OX-loaded Se-MnP NPs significantly inhibited proliferation of OX-resistant HCT116 (HCT116/DR) tumor cells in nude mice.

**Conclusions:**

OX@Se-MnP NPs with simple preparation and biomimetic chemical properties represent promising candidates for the treatment of colorectal cancer with MDR.

**Supplementary Information:**

The online version contains supplementary material available at 10.1186/s12951-023-01819-0.

## Introduction

Colorectal cancer (CRC) is the commonest malignancy of the digestive system with a high mortality rate[1]. According to data released by the World Health Organisation's International Agency for Research on Cancer (WHO IARC) in 2020, colorectal cancer is the second leading cause of cancer-related deaths with the third highest incidence of all cancers globally [2, 3]. Poor dietary habits, reduced activity, heavy alcohol consumption and smoking have been posited as causative factors for the increasing incidence of colorectal cancer [2]. Adjuvant chemotherapy is currently one of the commonly used treatments for non-metastatic colorectal cancer, with the combination of 5-fluorouracil, oxaliplatin (OX) and leucovorin used as a standard chemotherapy strategy [4–6]. OX-based chemotherapy plays an important role in the treatment of colorectal cancer [7–9]. However, the development of drug resistance is a major cause of chemotherapy failure in colorectal cancer [10, 11] leading to reduced chemotherapy efficacy and life expectancy [10, 12].

Multidrug resistance (MDR), the phenomenon of tumor resistance to numerous anticancer drugs, is a major barrier to effective chemotherapy [13] and associated with reduced curative rates and poor patient outcomes. The biological mechanisms underlying MDR and potential methods of overcoming MDR have been hot topics at the frontier of tumor pharmacology. Many therapeutic strategies aimed at overcoming MDR have been reported [14–17]. Unsurprisingly, sequential therapies and combinatorial therapies comprising several chemotherapeutic agents with non-overlapping mechanisms of action have proved effective in overcoming MDR[10, 18–21]. However, the efficacy of these two strategies has largely plateaued within recent decades and has been unable to completely overcome MDR [22, 23]. Further, novel anticancer immunotherapies (e.g., anti-CTLA4 and anti-PD-1/PD-L1) have also been shown to ultimately induce MDR at rates comparable to conventional chemotherapy [24, 25].

To date, a number of mechanisms have been posited to underlie multidrug resistance including increased drug efflux, increased DNA damage repair, reduced apoptosis, increased autophagy and altered drug metabolism [26–28]. Among them, ATP binding cassette (ABC) transporters serve as efflux pump of active drugs, and its overexpression is considered to be the main mechanism leading to reduced intracellular drug accumulation and drug resistance[29, 30]. Although several ABC transporter inhibitors have been reported, their clinically use has been limited due to concerns regarding uncertain active mechanisms and potential interactions with other chemotherapy drugs [31–33]. Accordingly, there is an urgent clinical need for the development of effective drug delivery systems that increase the uptake of chemotherapy drugs at tumor sites and potentially reverse tumor MDR [34].

Nanostructured drug delivery systems (NDDSs) have modifiable chemical properties and size, rapid clearance and the ability to sustain or control drug release and are considered promising candidates as therapies for reversing MDR in colorectal cancer cells [35]. Compared to traditional drug delivery system, NDDSs have many advantages including enhanced permeability and retention (EPR), biodegradability, biocompatibility and pH sensitivity. Many NDDSs are manufactured using components found in body tissues to avoid potential hazards and toxicity [36]. The payload and effective endocytosis can be controlled by adjusting the constituents and structure of NDDSs to avoid premature drug release and allow targeted drug delivery.

**Scheme 1:**
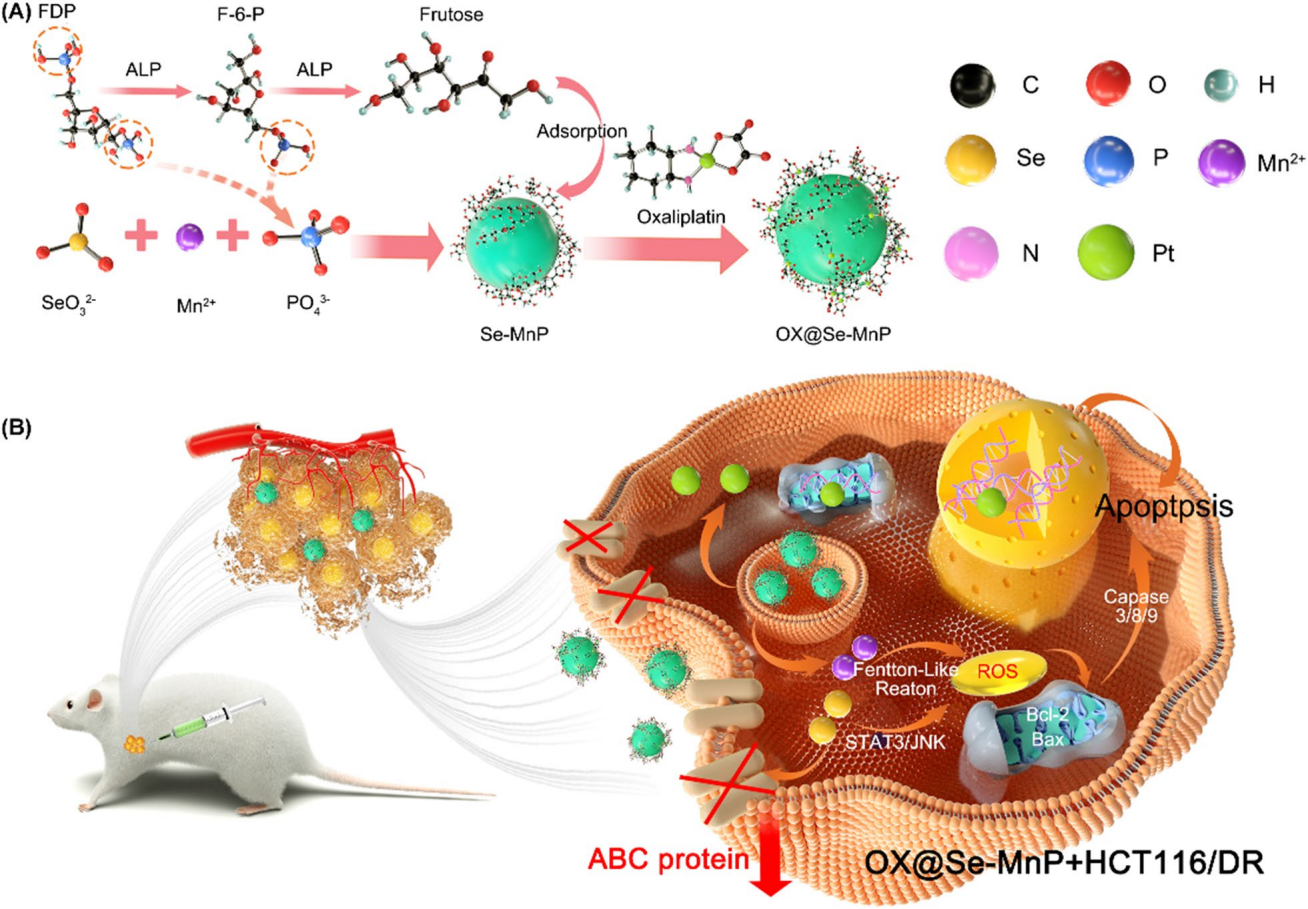
The scheme illustrates the synthetic procedure and the mechanism of antitumor induced by reversing drug resistance of nanomaterials.** A** Scheme illustration of the formation process and mechanism of the as-prepared OX@Se-MnP under the enzymatic reaction by using FDP as a phosphorus source.** B** Schematic illustration for the effect of OX@Se-MnP to reverse MDR of HCT116/DR cell.* FDP* Fructose-1,6-diphosphate,* F-6-P* Fructose-6-phosphate,* ALP* Alkaline phosphatase

The present study investigated the synthesis of selenium-doped manganese phosphate (Se-MnP) nanoparticles (NPs) with an amorphous structure using an alkaline phosphatase (ALP)-catalysed biomimetic strategy. The efficacy of Se-MnP NPs as nanocarriers for OX in the treatment of drug-resistant colorectal cancer was examined. The OX-resistant colorectal cancer cell line (HCT116/DR) and its parent strain (HCT116) were used to investigate the efficacy of OX@Se-MnP NPs in reversing MDR. The release of Se from OX@Se-MnP after being endocytosis by tumor cells was shown to down-regulate ABC transporters, which is known to function in transporting OX molecules from intracellular to extracellular spaces. Se and Mn^2+^ have promote excessive ROS production through both the STAT3/JNK pathway and Fenton-like reactions, respectively, thereby inducing mitochondrial damage. The down-regulation of ABC protein and mitochondrial damage lead to the reversal of tumor drug resistance, thereby leading to a synergistic effect in drug-resistant colorectal cancer (Scheme 1).

## Materials and methods

### Preparation of Se-MnP and MnP

The nanoparticles of Se-MnP were synthesized by an enzymatic reaction, using an organic phosphorylated molecules of Fructose disodium diphosphate (C_6_H_12_Na_2_O_12_P_2_, Na_2_FDP) (Sigma-Aldrich Co., US) to instead traditional phosphate salts. In a typical process in synthesis of Se-MnP, 0.66 g of Na_2_FDP was dissolved in 40 mL of deionized water, 0.02 g of Na_2_SeO_3_ (Sigma-Aldrich Co., US) was dissolved in 40 mL of deionized water, separately. Then, the above solutions were mixed and stirred for 5 min at 37 °C within a water bath. Thereafter, 1 mL of ALP aqueous solution (20 U/mL) was added to the mixed solution and magnetically stirred. Then, 0.19 g of MnCl_2_·4H_2_O (Aladdin Biochemical Technology Co., China) dissolved in 20 mL of water was dropwise added into the above solution. During the whole reaction process, the pH value of the reaction solution was kept between 8.0 and 8.5 by continually adding a dilute NaOH aqueous solution (0.5 M). Finally, the as-prepared samples were collected by centrifugation and washed with ethanol twice, deionized water twice, and freeze-dried into powders for further use. The MnP without Se element was used as a control sample. The synthesis procedure of the MnP sample is as follows: 0.66 g of Na_2_FDP was dissolved in 80 mL of deionized water and then mixed with 1 mL of ALP (20 U/mL) aqueous solution. The rest of process is same to the preparation of Se-MnP sample.

### Drug loading

The typical drug loading experiment was performed as follows: the dried powder (10 mg) of the as-prepared Se-MnP was dispersed in 5 mL solution with 10 mg OX. The suspension was treated in an ultrasonic bath for 1 h, and then shaken in a sealed vessel at 37 °C for 24 h, followed by centrifugation and drying to obtain the Se-MnP drug delivery system.

### Characterization

X-ray powder diffraction (XRD) patterns were recorded using an X-ray diffractometer (Rigaku D/max 2550 V, Cu Kα radiation, λ = 1.54178 Å). Fourier transforms infrared (FTIR) spectra were obtained with an FTIR spectrometer (FTIR-7600, Lambda Scientific, Australia). Scanning electron microscopy (SEM) was performed with a scanning electron microscope (Hitachi S4800, Japan), while Transmission electron microscopy (TEM) was carried out with a field-emission transmission electron microscope (FEI Talos F200X G2, USA). Thermogravimetric (TG) analysis was carried out with a simultaneous thermal analyzer (STA-409/PC, Netzsch, Germany) at a heating rate of 10 °C min^−1^ under flowing air.

### ·OH generation by Mn^2+^-mediated Fenton-like reaction

Methylene blue (MB, 10 µg/mL), H_2_O_2_ (10 mM), and Se-MnP (400 μg/mL) were reacted at 37 °C for 30 min. The UV absorption spectrum of the sample was measured with a UV spectrophotometer (UV-2600, SHIMADZU, Japan) to verify that the ·OH generated by the Fenton-like reaction induced MB degradation.

### Release behavior of OX@Se-MnP

20 mg of powder of the as-prepared sample was dispersed into 60 mL phosphate buffered saline (PBS) solution at pH 5.5 and 7.4, respectively. Thereafter, the solutions were shaken in sealed vessels at a constant shaking rate (100 rpm). The medium (5 mL) was withdrawn for analysis by Inductively Coupled Plasma Optical Emission Spectrometer (Agilent 730-OES, America) at given time intervals and replaced with the same volume of fresh PBS solution.

### Statistical analysis

All the data were represented as means ± standard deviation. Statistical analysis was performed using unpaired Student’s t-tests for two groups and one-way analysis of variance (ANOVA) or two-way ANOVA (GraphPad Prism software) for multiple groups, with all data points showing a normal distribution. To compare two non-parametric data sets, a Mann–Whitney U-test was employed. In all cases, differences were considered statistically significant if p < 0.05 (*p < 0.05, **p < 0.01, ***p < 0.001 and ****p < 0.0001).

### Cell culture

Human Intestinal Epithelial Cells (HIEC) and Human colorectal adenocarcinoma cells (HCT116), purchased from American Type Culture Collection, were cultured in 1640 medium containing 10% fetal bovine serum, 100 units/mL penicillin, and 0.1 mg/mL streptomycin, and the medium was changed every 2 days. The cell line of HCT116 was obtained from American Type Culture Collection (USA). While, the OX-resisted cell line of HCT116/DR was obtained from Shanghai Meixuan Biotechnology Co., Ltd., which was establishment on HCT116 (American Type Culture Collection, USA) by a drug-induced method with gradually increased OX concentration. The basic principle of building drug-resistant cell lines in vitro by increasing drug concentration method is that long-term contact with low-dose drugs causes changes in the drug chemical process of the cell itself, and the appearance of Pgp glycoprotein (P-glycoprotein) on the cell membrane makes the cell gradually tolerant to drugs [37, 38].

### Cell viability assay

HCT116 cells or OX resistant HCT116/DR cells (HCT116/DR) were seeded at a density of 8 × 10^3^ cells/well in 96-well plates and cultured for 24 h. OX (1-400 μg/mL), Se-MnP (1-400 μg/mL), and OX@Se-MnP (1-400 μg/mL) of different concentrations were added to each well and incubated for 24 h and 48 h. OX was used as a positive control, and a pure medium without adding any compounds was designed as a blank control. After 24/48 h, each well was added with 10% Cell Counting Kit-8 (CCK-8) solution containing to stain the living cells. CCK-8 is an indicator of REDOX reactions. In the presence of electron carrier 1-Methoxy PMS, dehydrogenase in living cells is used to catalyze the tetracycline salt WST-8 to generate a water-soluble formazan dye, and the amount of formazan dye production is in a linear relationship with the number of living cells. Then, the cells were placed in an incubator for 3 h (5% CO_2_, 37 °C). Finally, the optical density value was measured by a microplate reader (Eon, BioTek, U.S.A.) at a wavelength of 450 nm, and the results were then statistically analyzed and plotted. The cell viability was determined according to the equation:

$${\text{Cell viability }}\left( \% \right) \, = \left[ {\left( {{\text{As}} - {\text{Ab}}} \right)/\left( {{\text{Ac}} - {\text{Ab}}} \right)} \right] \, \times {100}\% ,$$

As: absorbance of experimental hole (including cells, medium, CCK-8 solution and drug solution); Ac: control hole absorbance (including cells, culture medium and CCK-8 solution, without drugs); Ab: blank hole absorbance (including culture medium and CCK-8 solution, but not cells and drugs). To ensure the validity of the experimental data, three repeated tests were performed on all samples.

### Staining for live/dead cells

HCT116/DR cells (2 × 10^5^) were seeded into a 6-well plate and were left to grow 12 h. The HCT116/DR cells were treated using PBS, OX (20 μg/mL), Se-MnP (100 μg/mL) and OX@Se-MnP (120 μg/mL), and incubated for 24 h. Calcein-AM/propidium iodide staining reagents were used to stain the viable and dead cells. After different treatments, the media in the 6-well plate was replaced with 1 mL 0.2 μM Calcein-AM/propidium iodide for 45 min coincubation. Afterward, fresh media was added to replace the Calcein-AM/propidium iodide solution for another 45 min at 37 °C. Finally, the cells were observed using a fluorescence microscope.

### ROS generation

ROS generation was measured by intracellular conversion of dichlorofluoresce in diacetate (DCFH/DA) into a fluorescent product DCF. HCT116/DR cells were plated in 6-well plates at 1 × 10^6^ cells/well density. After culturing for 24 h, the cells were separately incubated of OX (20 μg/mL), Se-MnP (100 μg/mL) and OX@Se-MnP (120 μg/mL) for 12 h. After that, DCFH/DA (1 μM) (Beyotime, China) was added to each well and incubated at 37 °C for 30 min before the cells were detached. The cells were subsequently collected by centrifugation, washed twice with PBS, and resuspended in PBS. Then, the generation of ROS in cells of different groups was observed under a Laser Confocal Scanning Microscopy (LCSM, FluoViewFV1000, Olympus, Japan).

### Cellular uptake

The cellular uptake of Se-MnP nanoparticles after being labeled with FITC was studied by LCSM. Firstly, Se-MnP (0.2 mg/mL) was mixed with FITC (10 μg/mL) in PBS, and the mixed solution was shaken (120 rpm) at 37 °C for 24 h. Then, the FITC-labeled Se-MnP nanoparticles were washed with PBS for two times and centrifugally separated. Finally, FITC-labeled Se-MnP nanoparticles (final concentration, 120 μg/mL) were added into the medium of HCT116/DR cells, which were cultured in confocal dishes for 24 h with a seeding density of 1 × 10^4^ cells/dish. After coculturing for 2, 4, 8 and 10 h, the cells were washed with PBS, fixed with 4% paraformaldehyde for 20 min, and stained with 10 μg/mL DAPI solution. Thereafter, the treated cells were observed in confocal dishes under LCSM.

### Western blotting

HCT116/DR cells were separately treated with OX of 20 μg/mL, Se-MnP of 100 μg/mL, and OX@Se-MnP of 120 μg/mL to analyze apoptotic proteins at 24 h. The cells were lysed for 30 min on ice, centrifuged at 12,000 rpm at 4 °C. The BCA assay was performed to measure the protein concentrations. After being mixed with the loading buffer and boiled, the same content of protein was loaded into SDS–polyacrylamide gel, separated by electrophoresis at 80 V for 40 min, then transferred to polyvinylidene fluoride (PVDF) membranes. After blocking with TBST (0.1% Tween-20, 10% tri-sec-buffer saline, 89.9% deionized water) containing 5% nonfat milk, these membranes were incubated with primary antibodies overnight at 4 °C, then incubated with HRP-conjugated secondary antibodies. The immunoreactive blots were observed using the ECL kit (Thermo Scientific Pierce, U.S.A.) with a chemiluminescence system (Bio-Rad). The Additional file 1: Table S2 shows all primary and secondary antibodies used in this article.

### Construction of animal model

The colorectal cancer model was constructed using BALB/c nude mice (4-6 weeks) to investigate antitumor activities of Se-MnP and OX@Se-MnP in vivo. All procedures involving animals were reviewed and approved by the Institutional Animal Care and Use Committee of Shanghai Tenth People’s Hospital (Number: SHDSYY-2021-4443). The colorectal cancer model was inoculated at the left shoulder by injecting 5 × 10^6^ cells/site of HCT116/DR cells. Fourteen days after cell implantation, the tumor volume was approximately 100 mm^3^. The mice were randomLy divided into 4 groups (5 mice per group), including OX (4 mg/kg), Se-MnP (16 mg/kg), OX@Se-MnP (20 mg/kg), and the blank control of physiological saline. The mice were intratumor injected with 100 μL of nanoparticles and PBS for each mouse.

### Evaluation of anti-colorectal cancer activity in vivo

The mice were repeatedly treated and weighed every 2 days, and the tumor volume was measured with a vernier caliper according to the equation: volume (mm^3^) = (length × width^2^)/2. On day 14, all mice were euthanized, and an autopsy was conducted. The tumors were detached, weighed, and fixed with 10% neutral formalin. Tumor tissues were embedded with paraffin, sectioned at a thickness of 5 μm, and mounted onto slides for histological examination. Tumor growth status and organ tissue lesions were observed using HE staining. Ki-67 assay was used to detect the proliferation of colorectal cancer cells in tumor tissues. The cleaved caspase-3 was investigated by immunohistochemical analysis according to a previously reported method [39]. The expression of MDR protein ABCB1, ABCC1, and ABCG2 were investigated by immunohistochemical analysis according to a previously reported method.

## Results and discussion

### Synthesis and characterisation

Scheme 1A illustrates the preparation and synthesis of Se-MnP via an enzymatic reaction-inspired strategy using fructose-1,6-diphosphate (FDP) as a phosphorus source instead of traditional phosphate salts. FDP can be gradually hydrolysed to fructose-6-phosphate (F-6-P), fructose and dissociative PO_4_^3−^ ions in an aqueous solution in a reaction catalysed by alkaline phosphatase (ALP). Free Mn^2+^ ions are able to react with hydrolysed PO_4_^3−^ and SeO_3_^2−^ ions to form a precipitate of Se-MnP clusters. In this process, FDP and its dephosphorylated products integrate with the primary Se-MnP clusters to prevent the crystallisation of clusters, with Se-MnP NPs obtained by assembly or aggregation of these clusters

**Fig.1:**
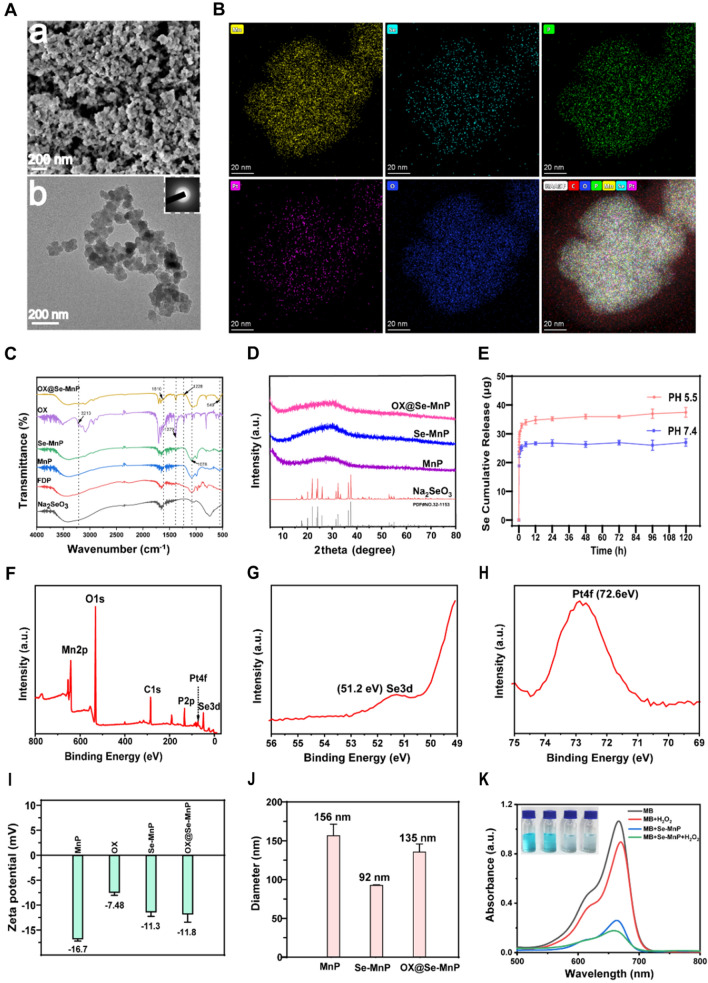
Synthesis and characterization of OX@Se-MnP. **A** SEM (**a**) and TEM (**b**) of Se-MnP. **B** Elemental mapping and corresponding merged images of OX@Se-MnP revealing the uniform distribution of Mn, Se, P, Pt and O elements in the matrix. **C** FTIR spectra of Na_2_SeO_3_, FDP, MnP, OX and Se-MnP before and after drug loading. **D** XRD patterns of Na_2_SeO_3_, MnP, Se-MnP and OX@Se-MnP. **E** The cumulative amount of Se released from OX@Se-MnP in PBS with different pH values (7.4 and 5.5). **F** The survey XPS spectrum of OX@Se-MnP. **G**, **H** XPS spectrum of Se 3d and Pt 4f in the OX@Se-MnP. **I** Zeta potentials of MnP, OX, Se-MnP and OX@Se-MnP. **J** Hydrodynamic diameter distribution of MnP, Se-MnP and OX@Se-MnP. **K** UV–visible absorption spectra and photo (inset) of MB degradation

In the present study, Se-MnP NDDSs were synthesised using a sustainable, simple and efficient enzyme-based catalytic method using FDP and Na_2_SeO_3_ as anion sources. FDP biomolecules and their dephosphorylated products have excellent biocompatibility as their naturally found in the human body. After the reaction, FDP biomolecules are integrated into MnP and Se-MnP to control their structures and crystal phases. Micrographs of Se-MnP from SEM and TEM are shown in Fig. 1A, demonstrating a NP structure with diameters of approximately 50-100 nm. MnP particles without elemental Se were used as a control (Additional file 1: Fig. S1). We then characterised the chemical and crystal properties of Se-MnP NPs by Fourier transform infrared (FTIR) spectra and X-ray diffraction (XRD). The presence of FDP and associated derivatives are revealed by FTIR spectra. The observance of absorption peaks at 1078 and 549 cm^−1^ can be attributed to the asymmetric stretching vibration of PO_4_^3−^ groups. The elemental mapping of manganese (Mn), selenium (Se), phosphorus (P), platinum (Pt) and oxygen (O) elements confirmed the successful synthesis of OX@Se-MnP with good dispersion of elemental Se, these carbons come from fructose and OX and the Pt comes from OX (Fig. 1B). The spectra peaks at 1228, 1379, 1610 and 3213 cm^−1^ associated with OX were also seen in the spectra of OX@Se-MnP NPs, indicating successful OX loading. These results demonstrate the preparation of Se-MnP NPs as inorganic-organic complexes. (Fig. 1C). XRD patterns of Se-MnP and OX@Se-MnP NPs revealed a broad hump at the 2θ value of approximately 30° with no further diffraction peaks indicating an amorphous crystal structure (Fig. 1D).

Next, we investigated the selenium and OX drug release kinetics of OX@Se-MnP in PBS buffers with different pH levels of 7.4 and 5.5. Normal healthy tissue has a pH close to 7.4, whereas tumor tissue or tumor cell microenvironment are acidic (~ 5.5). When the release time reached 12 h, the accumulation of Se from OX@Se-MnP was nearly 37 μg in the acidic condition and nearly 25 μg in the neutral condition (Fig. 1E). The cumulative release of OX was close to 50% under acidic conditions and close to 30% under neutral conditions (Additional file 1: Fig. S2). The result indicated that the release kinetics of OX from OX@Se-MnP NPs is a complex process, which is mainly attributed to a diffusion process. Meanwhile, this drug delivery system also displayed a pH responsive characteristic.

The valence state of OX@Se-MnP NPs was analysed by X-ray photoelectron spectroscopy, which clearly demonstrated manganese, selenium and platinum signals (Fig. 1F). The binding energy of Se 3d at 51.2 eV corresponds to metallic selenium (Se^0^) (Fig. 1G) [40] and the Pt 4f peak at 72.6 eV can be attributed to PtO (Fig. 1H), further confirming the presence of OX molecules in OX@Se-MnP NPs. As shown in Fig. 1I, the zeta potential changed from − 11.3 mV to − 11.8 mV after loading with OX due to owing the negative potential of OX (− 7.48 mV). Dynamic light scattering measurements of Se-MnP NPs were approximately 92 nm (Fig. 1J), which is consistent with the size of the sample observed under TEM. After OX loading, the particle size of nanoparticles is slightly increased to 135 nm.

Thermogravimetric (TG) analysis of Se-MnP and OX@Se-MnP NPs are shown in Additional file 1: Fig. S3. After loading of OX molecules, the weight loss of OX@Se-MnP NPs was found to be higher than Se-MnP NPs, as expected. The 2.9% weight loss observed in the TG curve of OX@Se-MnP NPs is predominantly attributable to oxygenolysis of OX molecules. The stability of Se-MnP NPs in aqueous solution at pH values of 7.4, 6.5 and 5.0 for 24 h demonstrated good stability of samples in solutions with a high pH value and degradation in acid solutions with low pH values (Additional file 1: Fig. S4). In addition, the UV absorption spectrum of Se-MnP NPs demonstrated decreased absorption peaks at wavelengths of 650–700 nm attributed to the Mn^2+^ ions in of Se-MnP NPs, indicating the occurrence of Fenton-like reactions inducing the formation of ·OH and degradation of MB molecules (Fig. 1K) [41].

### Cytotoxicity and cytophagocytosis of OX@Se-MnP NPs

The cytotoxicity of OX and Se-MnP with respect to human colorectal cancer cells (HCT116) and OX-drug-resistant HCT116 (HCT116/DR) cells were evaluated using a CCK-8 assay (Additional file 1: Figs. S5, S6). In the 48h toxicity test, almost 90% of HCT116 cells were non-viable at OX concentrations greater than 15 μg/mL, while more than half of drug-resistant HCT116/DR cells remained viable. The IC_50_ (half maximal inhibitory concentration) of HCT116/DR was 20.71 times greater than that of HCT116 after 48 h (Additional file 1: Fig. S5; Table S2). At the same concentration, Se-MnP NPs were more toxic to drug-resistant cells (HCT116/DR) (IC_50_, 24.09) than the cells of HCT116 cells after 48 h (IC_50_, 37.35) (Additional file 1: Fig. S6; Table S2). IC_50_ values are summarised in Additional file 1: Table S2.

As a bifunctional alkylating agent, OX interferes with DNA replication and ultimately induces apoptosis of tumor cells [42–44]. Despite the successful application of OX-based chemotherapy for the treatment of colorectal cancer, OX is associated with serious side effects, such as neurotoxicity, due to non-specific uptake [45–47]. Moreover, OX is less effective against tumor cells with MDR. The Se-MnP NPs prepared in the present study are suitable for drug loading due to their small size and stable amorphous morphology. Therefore, we further investigated the properties of Se-MnP NPs as nanocarrier of OX drug. The drug loading (DL%) and encapsulation efficiency (EE%) for OX in Se-MnP NPs was calculated as 12% and 22%, respectively, according to the following formulae:

$$DL\%=\frac{\text{Weight of loaded drug}}{\text{Weight of NPs and drug}}*100\%$$

$$EE\%=\frac{\text{Weight of loaded drug}}{\text{Unprepared drug weight}}*100\%$$

**Fig. 2:**
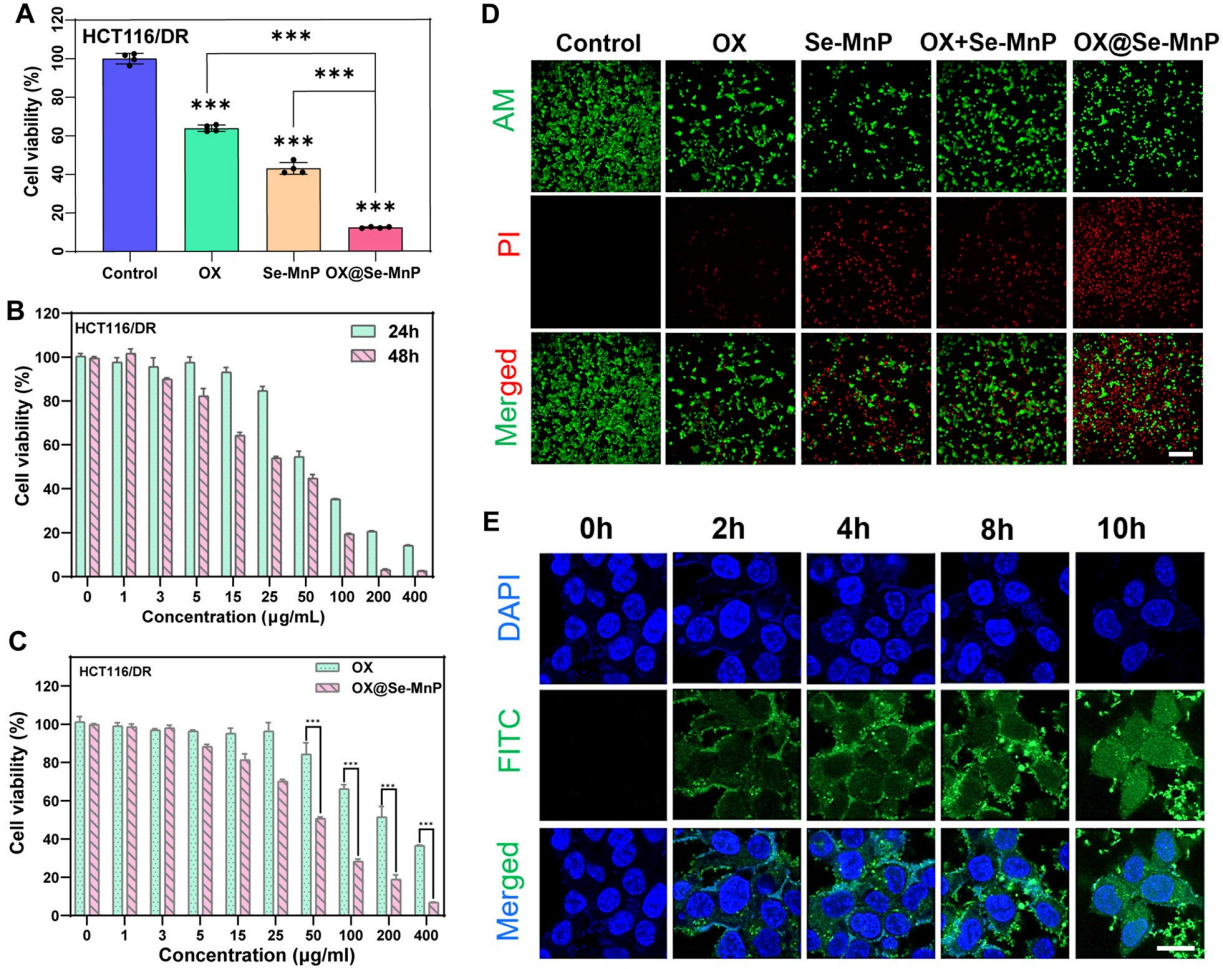
Cytotoxicity assessments of the OX@Se-MnP and uptake of FITC-labeled Se-MnP by HCT116/DR cells. **A** Relative cell viabilities of HCT116/DR cells co-incubation with OX (20 μg/mL), Se-MnP (100 μg/mL) and OX@Se-MnP (120 μg/mL) for 48 h. **B** Relative cell viabilities of HCT116/DR cells after 24 h and 48 h co-incubation with OX@Se-MnP at varied concentrations. **C** Relative cell viabilities of HCT116/DR cells co-incubation with OX and OX@Se-MnP at varied concentrations after 24 h. **D** The images of CAM and PI stained HCT116/DR cells treated with PBS, OX (20 μg/mL), Se-MnP (100 μg/mL) and OX@Se-MnP (120 μg/mL) for 48 h. (Green fluorescence: Calcein AM representing living cells, red fluorescence: PI representing dead cells). **E** FITC-labeled Se-MnPs were incubated with HCT116/DR cells. After incubating for 2 h, 4 h, 8 h and 10 h, the cells were stained with DAPI and were observed by fluorescence microscope. Scale bar: **D** 100 μm, **F** 10 μm.**p < 0.01 and ***p < 0.001

Co-culture of colorectal cancer cells with free OX, free Se-MnP NPs, or OX@Se-MnP NPs for 48 h demonstrated OX@Se-MnP NPs had superior cytotoxicity in HCT116/ DR cells compared to free OX and Se-MnP NPs (Fig. 2A). Free OX (20 μg/mL) and Se-MnP NPs (100 μg/mL) alone reduced the viability of HCT116/DR cells to approximately 63% and 42%, respectively. In contrast, the viability of HCT116/DR cells was reduced by nearly 90% in the OX@Se-MnP group, indicating a synergistic antitumor effect of OX and Se-MnP.

As shown in Fig. 2B, the cytotoxicity of OX@Se-MnP NPs was proportional to NP concentrations. After being co-cultured with OX@Se-MnP (> 50 μg/mL) for 24 h and 48 h, the viability of HCT116/DR cells was significantly decrease. As shown in Fig. 2C, OX@Se-MnP NPs lead to more significant cells damage than free OX alone under equivalent concentrations, indicating potential in reversing MDR. Figure 2D illustrates that PBS (control) and OX had minimal cytotoxicity against drug-resistant cells, while Se-MnP and OX@Se-MnP NPs induced greater levels of apoptosis in drug-resistant colorectal cancer HCT116/DR cells, consistent with the results of the CCK-8 assay. The images of Calcein AM and PI stained HCT116/DR cells treated with OX (10 μg/mL), Se-MnP (50 μg/mL), OX + Se-MnP and OX@Se-MnP (60 μg/mL) (Additional file 1: Fig. S7). LCSM demonstrated intracellular uptake of the FITC-labeled Se-MnP NPs as the green fluorescence of FITC-labelled NPs was observed inside cells (Fig. 2E), confirming that OX@Se-MnP NPs are taken up by HCT116/DR cells within two hours. This finding indicates OX@Se-MnP NPs could be rapidly up-taken by tumor cells, which may contribute to accumulating in tumor tissues in vivo*.*

### Mechanism of MDR reversal in HCT116/DR cells by Se-MnP

**Fig. 3:**
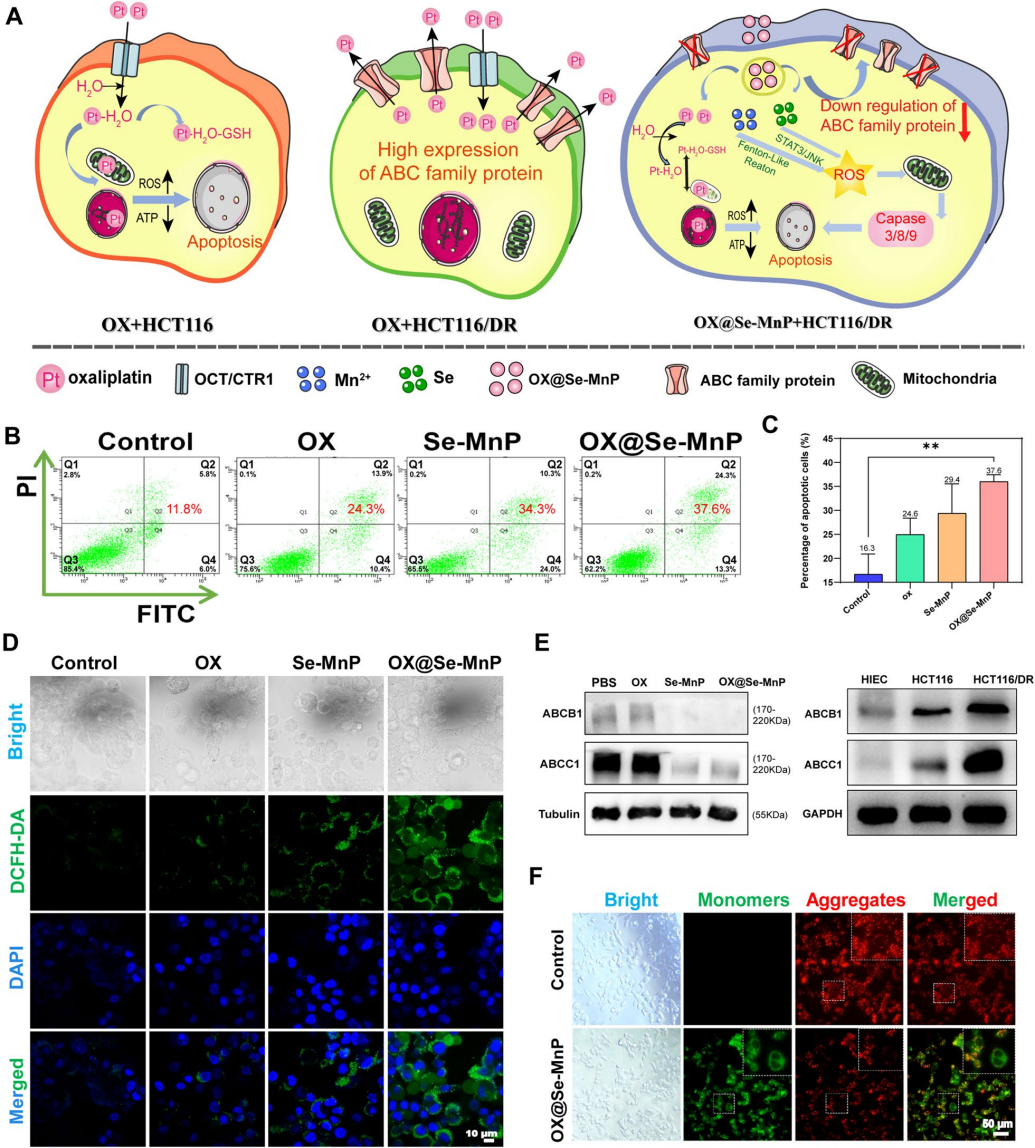
**A** The schematic representation of OX acts on drug-resistant and non-resistant cells, and OX@Se-MnP acts on drug-resistant cells. **B** Flow cytometric analysis of HCT116/DR cells at 24 h after various treatments including PBS, OX, Se-MnP and OX@Se-MnP. **C** The percentage of apoptotic cells was determined by flow cytometry. **D** Fluorescence images of HCT116/DR cells stained with DCFH-DA. **E** The expression levels of ABCB1 and ABCC1 protein. **F** Fluorescence images of HCT116/DR cells stained with JC-1.**p <0.01

A CCK-8 assay was used to evaluate the effects of Se-MnP NPs on drug resistance of HCT116 and HCT116/DR cells. First, the IC_50_ of OX was calculated to be 1.988 µg/mL and 41.17 µg/mL in HCT116 and HCT116/DR cells for 48 h, respectively (Additional file 1: Fig. S5; Table S2). Apoptosis rate was further assessed by flow cytometry, the control showed no obvious apoptosis rate. While the apoptosis of Se-MnP and OX@Se-MnP increased, the total % of apoptotic cells was calculated from the sum of Q2 and Q4 (Fig. 3B, C).

Reactive oxygen species are known to cause mitochondrial injury, thereby inducing apoptosis [48–50]. Therefore, apoptosis induced by overproduction of intracellular ROS was evaluated used the ROS fluorescence probe 2′,7′-dichloro luorescein diacetate (DCFH-A) as this probe is efficiently oxidised by ROS to emit green fluorescence. The use of ROS assay kits demonstrated that OX had little or no obvious detectable DCFH. However, the fluorescence intensity of DCFH was significantly enhanced in HCT116/DR cells after culture with Se-MnP and OX@Se-MnP NPs (Fig. 3D). In agreement with the CCK8 assay and flow cytometry, these results indicate that ROS overproduction induced by OX@Se-MnP NPs has a significant suppressive effect on cancer cells. Elemental Se and Se-containing compounds have been used clinically for cancer prevention and treatment as Se induces the production of cytotoxic levels of ROS in many cancer types including malignant mesothelioma [51], prostate cancer [52] and liver cancer [53]. The Se reportedly increases oxidative stress in tumor tissues and exerts antitumor activities including: inhibiting tumor growth, VEGF expression and metastasis; enhancing immune response and decreasing antitumor drug resistance [54]. In addition, Se has been shown to activate the STAT3/JNK pathway leading to increased ROS levels [55]. The results of the ROS assay indicate that ROS generation plays a vital role in the process of selenium-induced cell apoptosis (Fig. 3D), possibly mediated by caspase-8/caspase-3-dependent apoptosis in response to increased ROS levels [56]. Then, the expression levels of cleaved caspase-3, caspase-8 in HCT116/DR cells treated with OX, Se-MnP and OX@Se-MnP have been studied, and the result in Additional file 1: Fig. S8 further demonstrates the above hypothesis.

We further evaluated the effect of Se-MnP NPs on inhibiting ABC transporters, which play an important role in the MDR of cancer cells. According to previous studies, ABC transporters (ABCB1, ABCC1) are highly expressed in drug-resistant strains compared to parental strains [26, 30, 57, 58]. As representative members of the ABC family of proteins, the expression levels of ABCB1 and ABCC1 in HCT116/DR cells was significantly decreased by Se-MnP and OX@Se-MnP NPs. The results demonstrated that the ABC transporter (ABCB1, ABCC1) is highly expressed in drug-resistant strains compared to Human Intestinal Epithelial Cells (HIEC) and parent strains (Fig. 3E).

Mitochondrial damage may cause mitochondrial depolarisation leading to decreases in membrane potential. In the present study, the mitochondrial membrane potential (△Ψm) was measured using JC-1 fluorescence probes which shift from red to green fluorescence upon loss of △Ψm. As shown in Fig. 3F, OX@Se-MnP NPs induced dramatic loss of △Ψm, indicating OX@Se-MnP NPs likely induce mitochondrial dysfunction leading to ROS overproduction and subsequent cell apoptosis [40, 59]. Fluorescence image of JC-1 stained HCT116/DR cells co-incubated with OX and Se-MnP for 24 h (Additional file 1: Fig. S9).

### In vivo antitumor effect of NPs

**Fig. 4:**
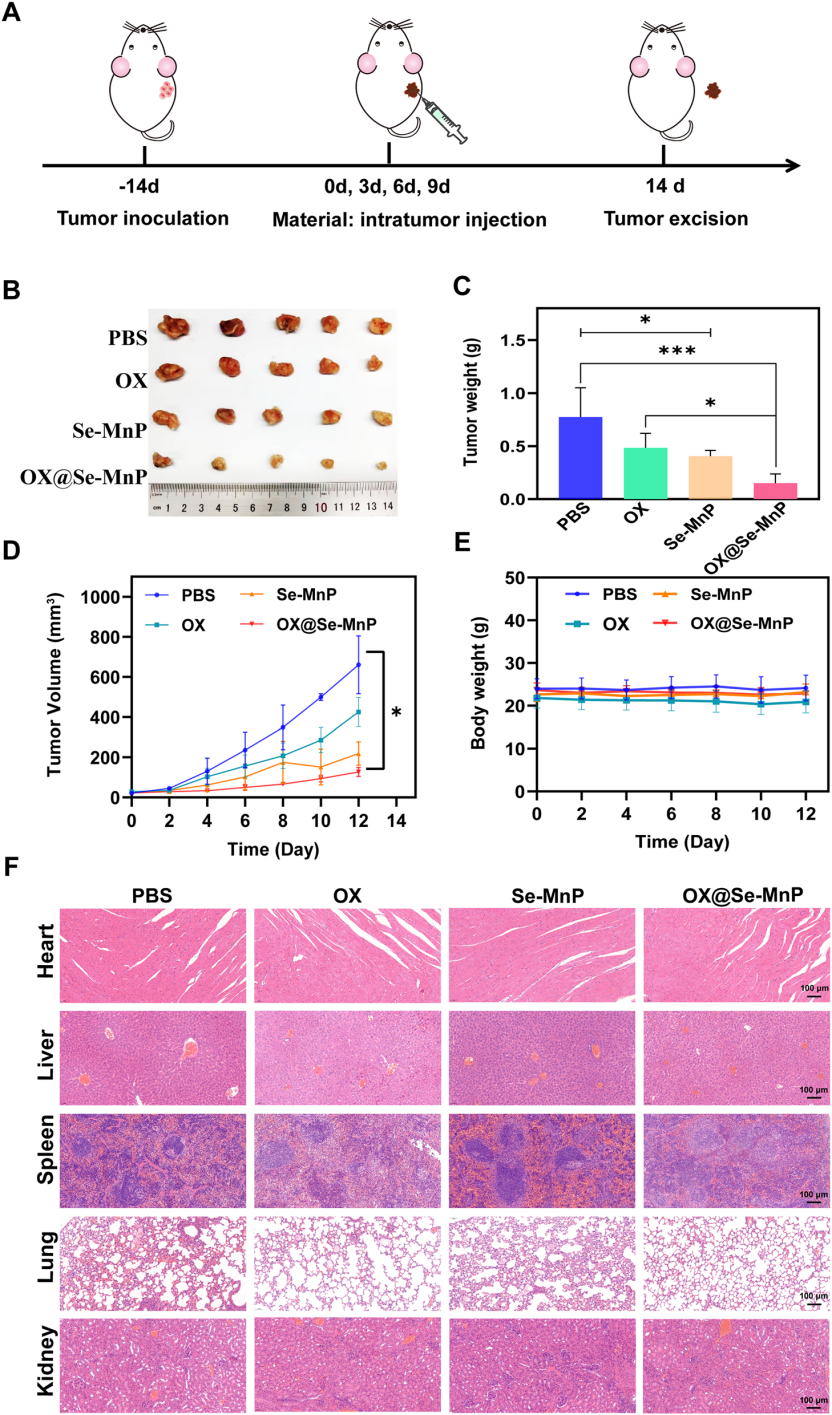
**A** Scheme illustration for the in vivo evaluation of antitumor therapy. **B** Photographic images of the tumor at the end of these treatments. **C** Average weights of tumors harvested at the end of these treatments (n = 5). **D** Relative tumor volume of mice after various treatments. **E** Average weights of mice. **F** Histological analysis of the main organs (heart, liver, spleen, lung and kidney) of untreated mice (PBS) and mice treated with OX, Se-MnP and OX@Se-MnP. *p < 0.05, ***p < 0.001

Finally, we investigated the in vivo antitumor efficacy and systemic toxicity of OX@Se-MnP NPs in BALB/c nude mice with subcutaneous MDR colorectal tumors (Fig. 4A). Mice in control groups received intra-tumoral administration of PBS or OX (4 mg/kg). Mice in treatment groups received intra-tumoral administration of Se-MnP (16 mg/kg) or OX@Se-MnP NPs (20 mg/kg) every three days to evaluate antitumor efficacy in vivo. At the end of the treatment period (day 14), mice treated with OX@Se-MnP NPs had significantly reduced tumor volumes (660 ± 140 to 120 ± 22 mm^3^) and weights (0.77 ± 0.27 to 0.15 ± 0.08 g) compared to the control group (Fig. 4B–D). At six days of treatment, mice administered OX@Se-MnP NPs had significantly reduced tumor growth compared to other groups, with the inhibitory effect of OX@Se-MnP NPs gradually becoming more apparent with increasing treatment time (Fig. 4D). Furthermore, OX@Se-MnP NPs had a significant inhibitory effect on tumor growth compared to OX alone and Se-MnP NPs. All mice survived to the end of experimental period. No differences in body weight were observed between mice treated with NPs and the control group, indicating NPs had good biocompatibility (Fig. 4E).

Further, heart, liver, spleen, lung and kidney specimens were sectioned for hematoxylin and eosin (H&E) staining to evaluate the toxicity of NPs. The images of H&E histochemistry from heart, liver and kidney demonstrated normal tissue morphology in the treatment groups with no observable organ injury (Fig. 4F). However, there are slight differences in the HE images of spleens and lungs from the groups treated with OX and OX@Se-MnP, compared with the control group. The difference may be resulted by the effect of OX, which is a normal cancer drug and has high cytotoxicity. Co-culture of drug-resistant colorectal cancer cells with free OX, Se-MnP NPs, or OX@Se-MnP NPs demonstrated that OX@SeMnP NPs had greater cytotoxicity than free OX or Se-MnP NPs. The anticancer effect of Se-MnP and the synergistic effect of Se and OX in OX@Se-MnP NPs were further confirmed by the in vivo experiment, with tumor volumes in the OX@Se-MnP NP group significantly smaller than in groups treated with Se-MnP NPs or OX alone.

**Fig. 5:**
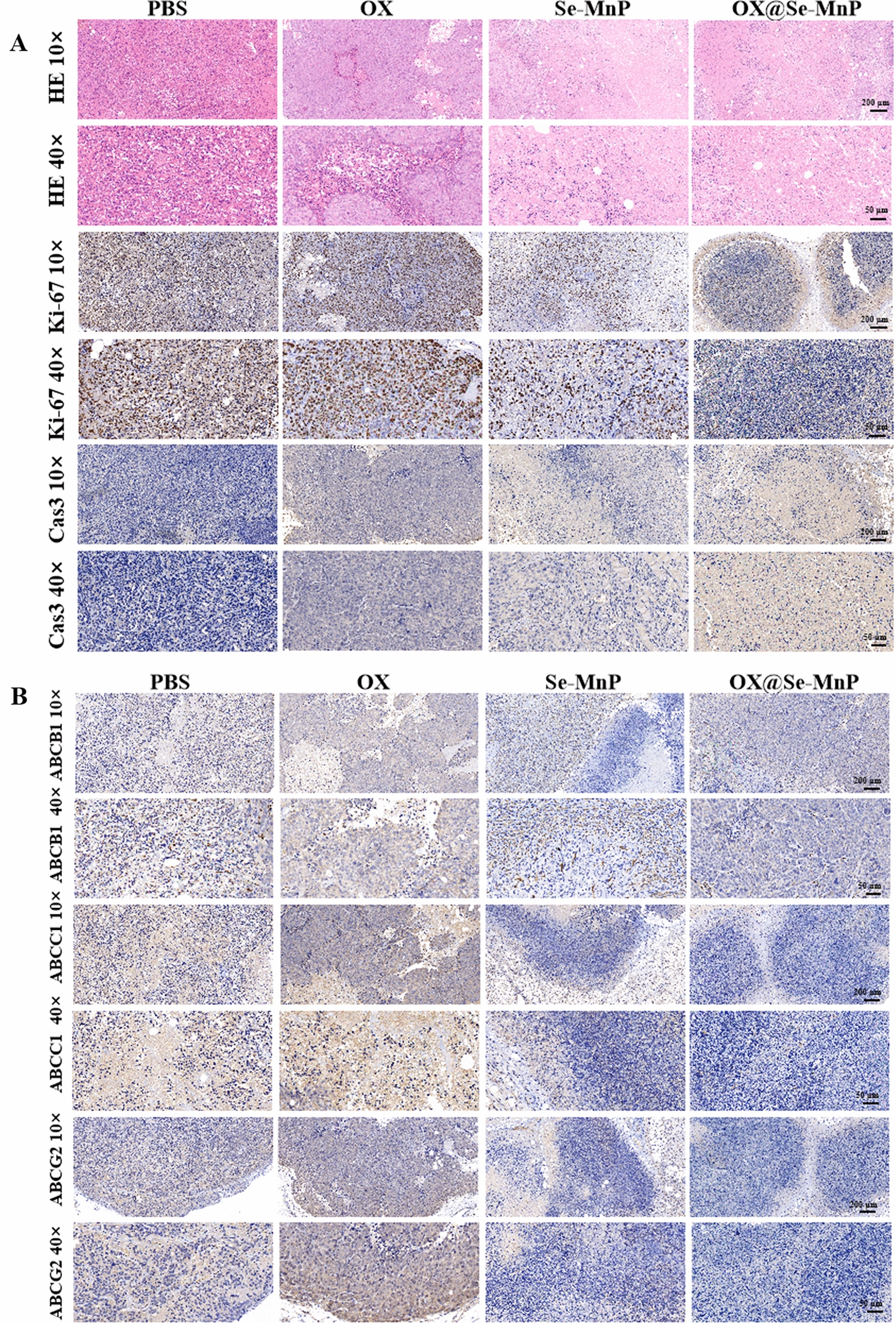
**A** Histological analysis of tumor tissues stained with hematoxylin and eosin, Ki-67, and cleaved caspase-3 from these groups. Tumors treated with Se-MnP and OX@Se-MnP exhibit typical apoptotic appearance (cell shrinkage, karyolysis and nuclear fragmentation), poor cell proliferation and higher cleaved caspase-3 expression, comparing with free PBS treatments. **B** Histological analysis of tumor tissues stained with ABCB1, ABCC1 and ABCG2 from these groups. The pathological analysis further demonstrated that tumors treated with Se-MnP and OX@Se-MnP exhibited lower expression of ABCB1, ABCC1 and ABCG2, compared with those treated with OX

H&E and immunohistochemical staining for Ki67, cleaved caspase-3, ABCB1, ABCC1 and ABCG2 were performed in tumor samples (Fig. 5). As shown in Fig. 5A, HCT116/DR tumor cells in the control group proliferated well with large, oval-shaped nuclei. Intra-tumoral administration of OX had little effect on tumor growth. In contrast, tumors treated with Se-MnP or OX@Se-MnP NPs had a typical appearance of apoptotic tumor tissue with condensed nuclear chromatin. Ki67 and cleaved caspase-3 expression were further analysed in tumor samples. Ki67 is a well-known marker for cellular proliferation, in the Se-MnP and OX@Se-MnP groups, decreased cell proliferation, Ki67 expression was markedly decreased.

The cleaved caspase-3 is a central regulator of apoptosis [60, 61]. In the Se-MnP and OX@Se-MnP groups, the expression of cleaved caspase-3 was significantly increased compared to the control group and promoting apoptosis by provoking the cleaved caspase-3 signaling pathway. These results demonstrate that Se-MnP and OX@Se-MnP NPs inhibit the proliferation of tumor cells and induce apoptosis of tumor cells via activation of the caspase-3 signalling pathway.

Expression levels of ABCB1, ABCC1 and ABCG2 were also examined in HCT116/DR tissues of mice treated with free OX, Se-MnP NPs, or OX@Se-MnP NPs. As shown in Fig. 5B, high expression levels of ABCB1, ABCC1 and ABCG2 were observed in the control groups and groups treated with free OX via local injection. However, reduced expression of ABCB1, ABCC1 and ABCG2 was observed in the Se-MnP and OX@Se-MnP groups, indicating the ability of Se-MnP NPs to reverse drug resistance in tumors by reducing the expression of ABC transporters.

In the present study, increased ROS levels lead to caspase-mediated apoptosis, as shown in Figs. 3 and 5. Furthermore, both in vitro and in vivo studies demonstrated that OX@Se-MnP NPs are taken up by colorectal cancer cells and continue to generate ·OH via a Fenton-like catalytic reaction, subsequently triggering caspase-mediated tumor apoptosis[62]. Thus, Se-MnP NPs function synergistically with OX to induce apoptosis in vitro and suppress tumor growth in vivo. These results indicate that OX@Se-MnP NPs represent an advanced NDDS system that may have efficacy in the treatment of cancer[63, 64].

## Conclusions

In summary, the present study demonstrates the synthesis of Se-MnP-based drug carriers with an organic–inorganic hybrid constituent and amorphous structure. NPs had efficacy in reducing MDR in tumor cells in vitro. Further, Se-MnP NPs increased generation of ROS and synchronously induced apoptosis in colorectal cancer cells. More importantly, treatment with Se-MnP NPs led to down-regulation of MDR-related ABC family proteins (ABCB1 and ABCC1) to reverse MDR in HCT116/DR tumor cells. Se-MnP NPs were able to efficiently encapsulate OX and synergistically induce DNA damage and apoptosis in HCT116/DR cells. Finally, in vivo studies further demonstrated that Se-MnP and OX@Se-MnP NPs reversed MDR in tumor cells and induced tumor apoptosis to suppress tumor growth by down-regulating the expression of MDR-related ABC transporters proteins (ABCB1, ABCC1 and ABCG2). The Se-MnP based drug carrier may represent a promising drug carrier for colorectal tumor therapy by synchronously inducing apoptosis and reversing MDR in tumor cells.

## Supplementary Information

**Additional file 1: Fig. S1.** Transmission electron microscopy (TEM) of MnP. **Fig. S2.** The cumulative amount of OX released from OX@Se-MnP NPs in PBS with different pH values (7.4 and 5.5). **Fig. S3.** Thermgravimetric analysis of Se-MnP and OX@Se-MnP. **Fig. S4.** Stability of Se-MnP at PBS solution with different pH values of 7.4, 6.5 and 5.0 for 24 h. **Fig. S5.** Viability and related IC50 of HCT116 and HCT116/DR cells treated with OX in different concentrations. (A-D) Viability and IC50 of HCT116 cells treated with OX for 24 (A,B) and 48 (C,D) hours, (E-H) Viability and IC50 of HCT116/DR cells treated with OX for 24 (E,F) and 48 (G,H) hours. **Fig. S6.** (A, B) Viability of HCT116/DR and HCT116 cells of treatment with Se-MnP with different concentrations. **Fig. S7.** The images of Calcein AM and PI stained HCT116/DR cells treated with OX (10 μg/ml), Se-MnP (50 μg/ml), OX+Se-MnP and OX@Se-MnP (60 μg/ml). **Fig. S8.** The expression level of cleaved caspase-3, caspase-8 in HCT116/DR cells treated with OX, Se-MnP and OX@Se-MnP. **Table S1.** All primary and secondary antibodies used in the paper. **Table S2.** Cytotoxic effects of Se-MnP and Oxaliplatin.

## Abbreviations

ABC: ATP binding cassette
EPR: Enhanced permeability and retention
H&E: Hematoxylin and eosin
NDDS: Nanostructured drug delivery systems
FDP: Fructose-1,6-diphosphate
F-6-P: Fructose-6-phosphate
ALP: Alkaline phosphatase
TEM: Transmission electron microscopy
XRD: X-ray diffraction
