# Supplementary material for: Enzyme-catalyzed synthesis of selenium-doped manganese phosphate for synergistic therapy of drug-resistant colorectal cancer
Source: J Nanobiotechnology. 2023 Mar 1;21:72. doi: 10.1186/s12951-023-01819-0 (PMC9976439; doi:10.1186/s12951-023-01819-0)
Supplement: Supplementary file 1 — Additional file 1: Fig. S1. Transmission electron microscopy (TEM) of MnP. Fig. S2. The cumulative amount of OX released from OX@Se-MnP NPs in PBS with different pH values (7.4 and 5.5). Fig. S3. Thermgravimetric analysis of Se-MnP and OX@Se-MnP. Fig. S4. Stability of Se-MnP at PBS solution with different pH values of 7.4, 6.5 and 5.0 for 24 h. Fig. S5. Viability and related IC50 of HCT116 and HCT116/DR cells treated with OX in different concentrations. (A-D) Viability and IC50 of HCT116 cells treated with OX for 24 (A,B) and 48 (C,D) hours, (E-H) Viability and IC50 of HCT116/DR cells treated with OX for 24 (E,F) and 48 (G,H) hours. Fig. S6. (A, B) Viability of HCT116/DR and HCT116 cells of treatment with Se-MnP with different concentrations. Fig. S7. The images of Calcein AM and PI stained HCT116/DR cells treated with OX (10 μg/ml), Se-MnP (50 μg/ml), OX+Se-MnP and OX@Se-MnP (60 μg/ml). Fig. S8. The expression level of cleaved caspase-3, caspase-8 in HCT116/DR cells treated with OX, Se-MnP and OX@Se-MnP. Table S1. All primary and secondary antibodies used in the paper. Table S2. Cytotoxic effects of Se-MnP and Oxaliplatin. [file 12951_2023_1819_MOESM1_ESM.docx]

**Supporting Information**

**Enzyme-****Catalyzed Synthesis of Selenium-Doped Manganese Phosphate for Synergistic Therapy of Drug-Resistant Colorectal Cancer**

Manman Pei^1,2†^, Kaiyuan Liu^2†^, Xiao Qu^2†^, Kairuo Wang^2^, Qian Chen^2^, Yuanyuan Zhang^2^, Xinyue Wang^2^, Zheng Wang^1,2^, Xinyao Li^2^, Feng Chen^1,2^*, Huanlong Qin^2^*, and Yang Zhang ^1,2,3^*

**Author affiliations:**

^1^School of Medicine, Anhui University of Science and Technology, 168 Taifeng Street, Shannan New District, Huainan city, Anhui Province 232000, P.R. China.

^2^Nanotechnology and Intestinal Microecology Research Center, Shanghai Tenth People’s Hospital, School of Medicine, Tongji University, Shanghai, 301 Yanchang Road, Shanghai 200072, P.R. China.

^3^Precision Medicine Center, Taizhou Central Hospital, 999 Donghai Road, Taizhou, Zhejiang Province 318000, P.R. China.

***Corresponding authors:**

fchen@tongji.edu.cn (Feng Chen)

qin_huanlong@126.com (Huanlong Qin)

zhangyang0202@tongji.edu.cn (Yang Zhang)


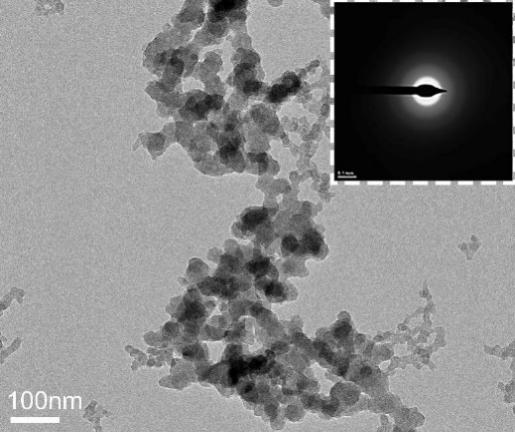


**Fig. S1.** Transmission electron microscopy (TEM) of MnP.

**Fig. S2.** The cumulative amount of OX released from OX@Se-MnP NPs in PBS with different pH values (7.4 and 5.5).

**
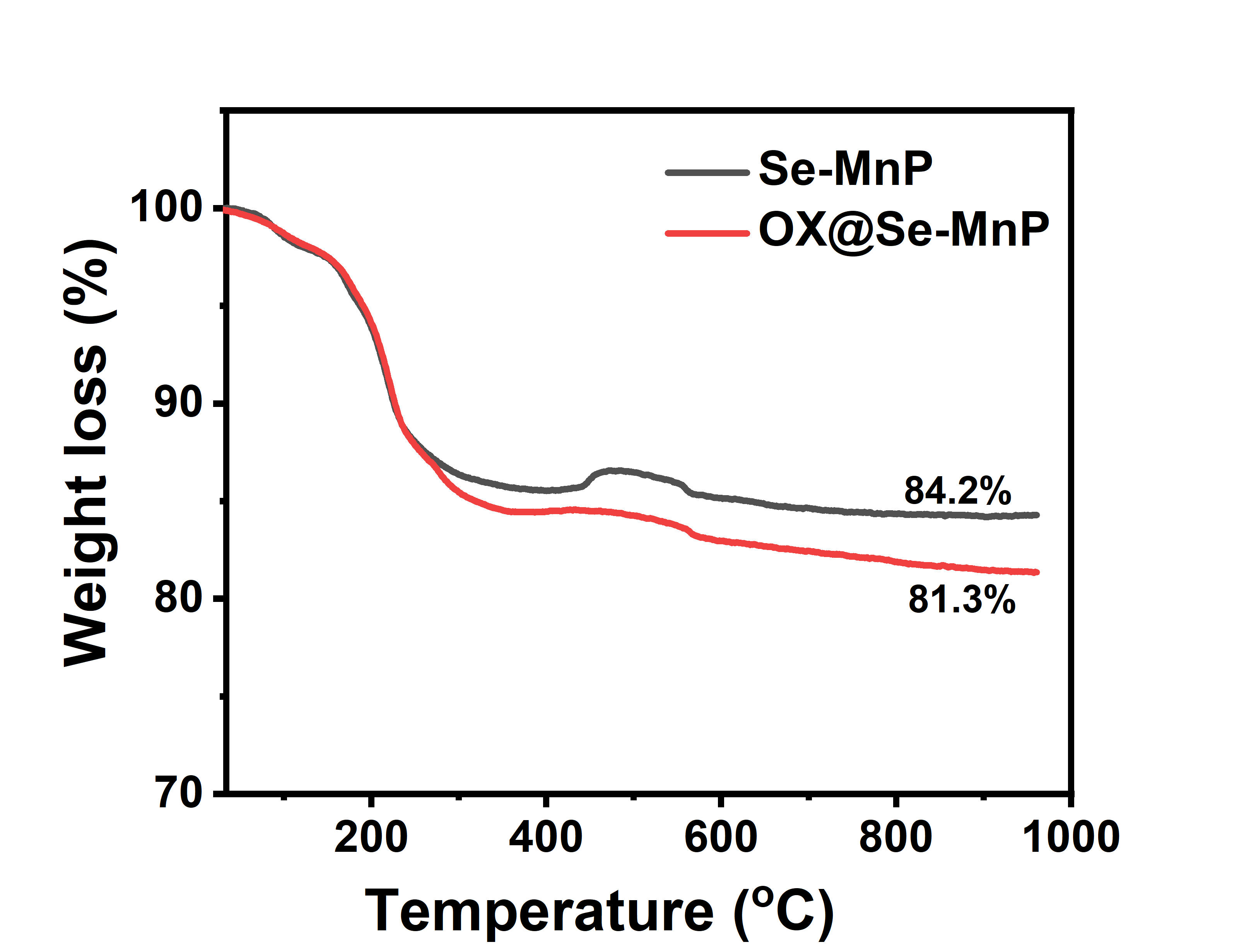
**

**Fig. S3.** Thermgravimetric analysis of Se-MnP and OX@Se-MnP.


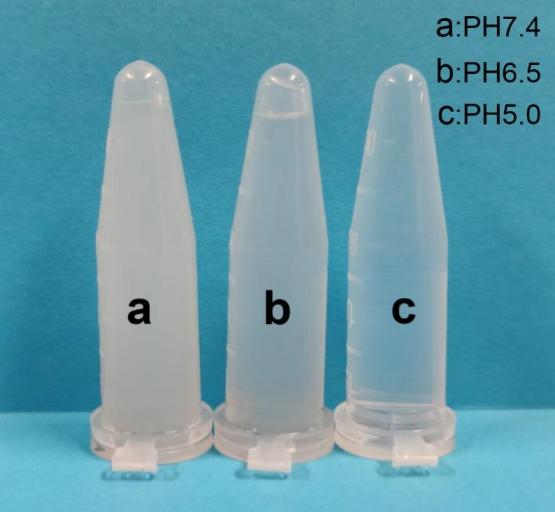


**Fig. S4****.** Stability of Se-MnP at PBS solution with different pH values of 7.4, 6.5 and 5.0 for 24 h.

**
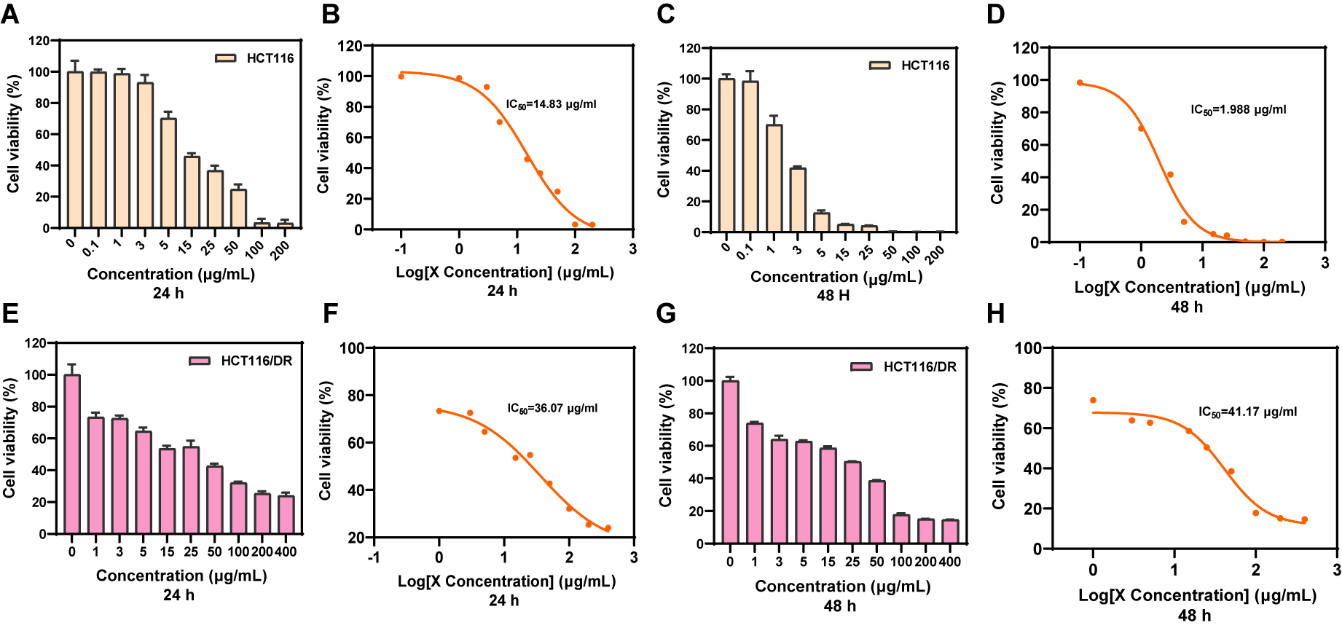
**

**Fig.S5.** Viability and related IC_50_ of HCT116 and HCT116/DR cells treated with OX in different concentration. **(A-D)** Viability and IC_50_ of HCT116 cells treated with OX for 24 (A, B) and 48 (C,D) hours, **(E-H)** Viability and IC_50_ of HCT116/DR cells treated with OX for 24 (E,F) and 48 (G,H) hours.


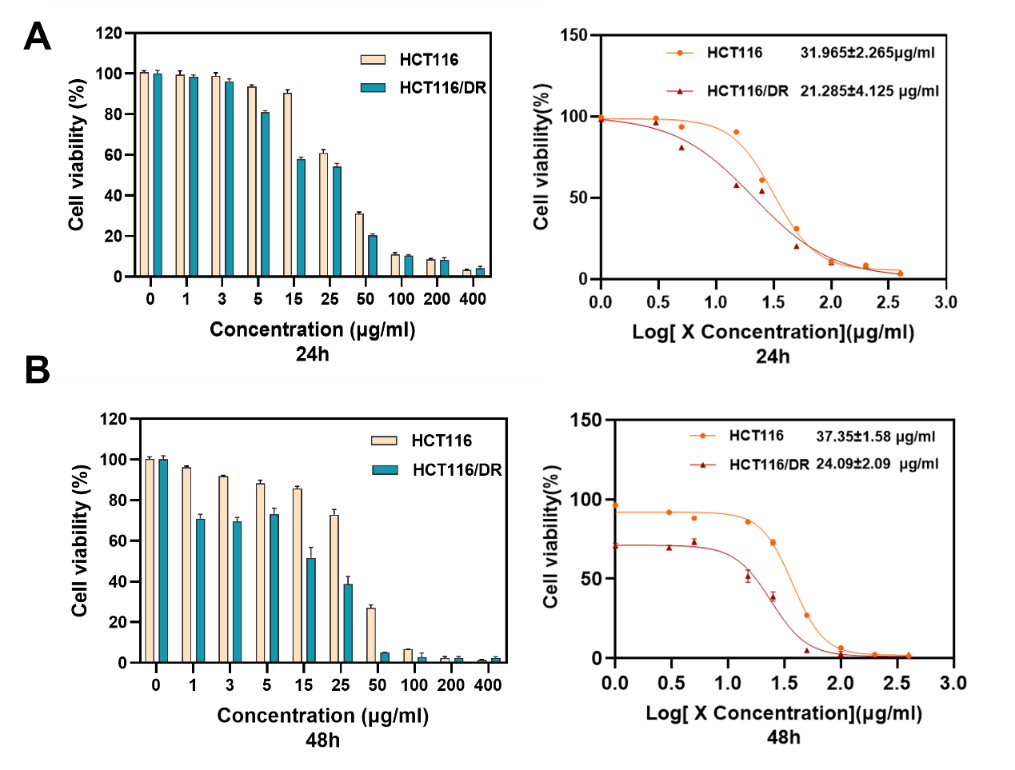


**Fig.S6. (A, B)** Viability of HCT116/DR and HCT116 cells of treatment with Se-MnP with different concentration.


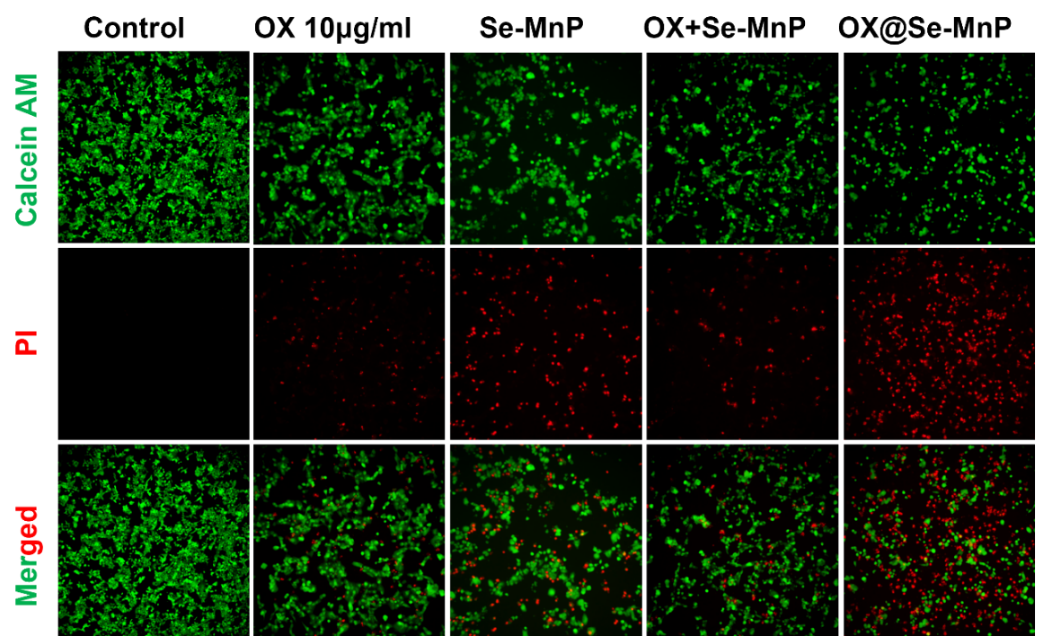


**Fig.S7.** The images of Calcein AM and PI stained HCT116/DR cells treated with OX (10 μg/ml), Se-MnP (50 μg/ml), OX+Se-MnP and OX@Se-MnP (60 μg/ml).


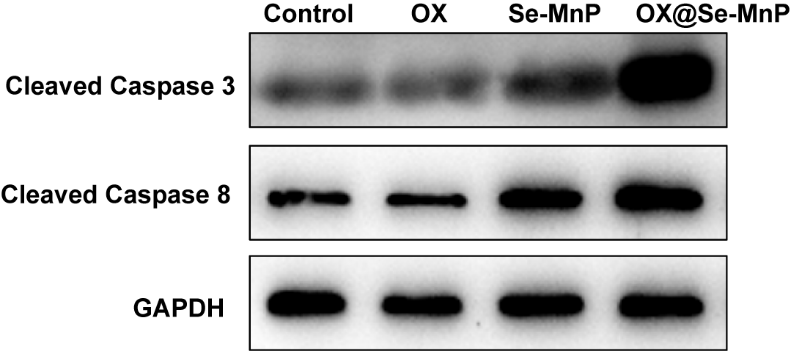


**Fig.S8.** The expression level of cleaved caspase-3, caspase-8 in HCT116/DR cells treated with OX, Se-MnP and OX@Se-MnP.


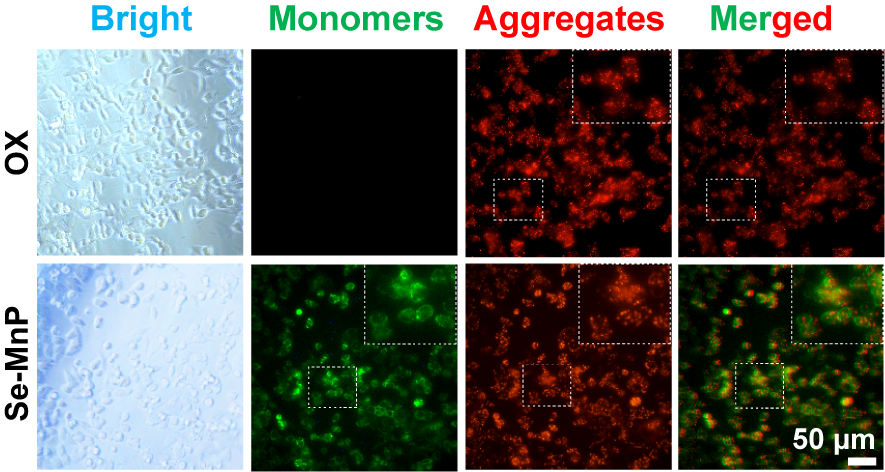


**Fig.S9.** Fluorescence image of JC-1 stained HCT116/DR cells co-incubated with OX and Se-MnP for 24h.


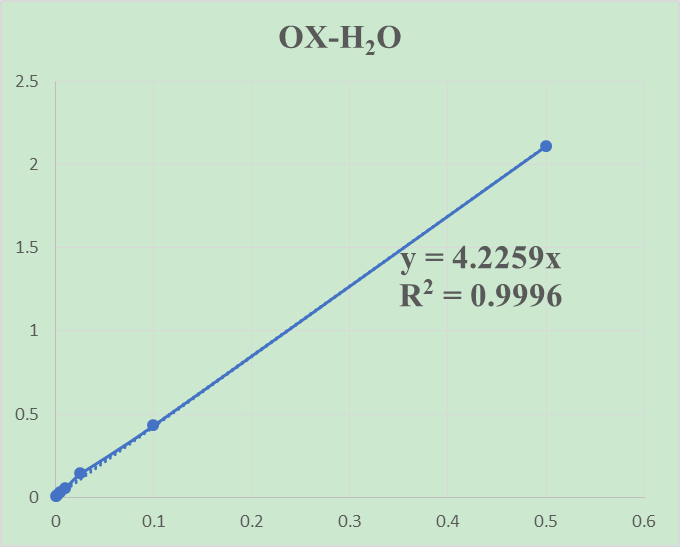

$$DL\%=\frac{Weight of loaded drug}{Weight of nanoparticle and drug}*100\%$$

$$EE\%=\frac{Weight of loaded drug}{Weight of feeding drug}*100\%$$

**Supplement Table**

**Table S1.** All primary and secondary antibodies used in the paper.

| Antibodies | | Commercial names | Catalog number |
| --- | --- | --- | --- |
| Primary antibodies | ABCB1 | MDR1/ABCB1 (E1Y7S) Rabbit mAb, E1Y7S, Cell Signaling | #13978 |
|  | ABCC1 | MRP1/ABCC1 (D5C1X) Rabbit mAb, D5C1X, Cell Signaling | #72202 |
|  | Anti-Cleaved Caspase 3 | Anti-Cleaved Caspase-3 antibody, ab2302，Abcam | #ab2302 |
|  | Anti-Cleaved Caspase 8 | [Cleaved Caspase-8 (Asp374) (18C8) Rabbit mAb](https://www.cellsignal.com/products/primary-antibodies/cleaved-caspase-8-asp391-18c8-rabbit-mab/9496), Cell Signaling | #9496 |
|  | Anti-Tubulin | α-Tubulin (DM1A) Mouse mAb, DM1A, Cell Signaling | #3873 |
|  | Anti-GAPDH | GAPDH (D16H11) XP® Rabbit mAb, D16H11, Cell Signaling | #5174 |
| Secondary antibodies | Anti-rabbit IgG | Anti-rabbit IgG, HRP-linked Antibody, Cell Signaling | #7074 |
|  | Anti-mouse IgG | Anti-mouse IgG, HRP-linked Antibody，Cell Signaling | #7076 |

**Table S2.** Cytotoxic effects of Se-MnP and Oxaliplatin.

| Materials | IC_50_ (μg/mL) | | |
| --- | --- | --- | --- |
|  | HCT116 | HCT116/DR | RI |
| Se-MnP (48 h) | 37.35 | 24.09 | 0.64 |
| OX (48 h) | 1.988 | 41.17 | 20.71 |

RI (resistance index): the ratio of IC_50_ (HCT116/DR) against IC_50_ (HCT116).
